# Effect of Chemical Enhancers in Transdermal Permeation of Alfuzosin Hydrochloride

**DOI:** 10.5402/2012/965280

**Published:** 2012-12-20

**Authors:** D. Prasanthi, P. K. Lakshmi

**Affiliations:** Department of Pharmaceutics, G. Pulla Reddy College of Pharmacy, Osmania University, Hyderabad, Andhra Pradesh 500 028, India

## Abstract

The objective of the present study is to explore the efficient chemical penetration enhancer among the various enhancers available in overcoming the stratum corneum barrier in transdermal delivery of Alfuzosin hydrochloride (AH). The different enhancers were incorporated in 2% Carbopol gel which was selected as a control and evaluated by in vitro diffusion studies through dialysis membrane and permeation through the rat abdominal skin using Keshary-Chien diffusion cells. All the enhancers increased the release rate through the dialysis membrane when compared with control except oleic acid which decreased the release rate but showed maximum solubility of the drug. Among the various enhancers Transcutol 20% and tween-20 (2%) showed the highest cumulative amount (*Q*_24_) of 702.28 ± 6.97 **μ**g/cm^2^ and 702.74 ± 7.49 **μ**g/cm^2^, respectively. A flux rate of 31.08 ± 0.21 **μ**g/cm^2^/hr by Transcutol 20% and 30.38 ± 0.18 **μ**g/cm^2^/hr by tween-20 (2%) was obtained. Transcutol 20% showed decreased lag time of 0.13 ± 0.05 hr. The lowest skin content of 342.33 ± 5.30 **μ**g/gm was seen with oleic acid 2.5%. Maximum enhancement of flux by 3.94-fold was obtained with transcutol 20%. Primary skin irritation studies were performed with rabbit. Histopathological studies of transcutol 20% showed marked changes such as degeneration and infiltration of mononuclear cells in dermis indicating the effect of transcutol on the skin. Among the different enhancers transcutol is efficient in enhancing transdermal delivery of AH.

## 1. Introduction

Alfuzosin hydrochloride (AH), the *α*-adrenoreceptor antagonist, is used for treating benign prostatic hyperplasia. It is indicated for long-term therapy in place of surgery. It undergoes extensive first pass metabolism, has a bioavailability of 60%, half-life (3–5 hour), log⁡⁡*P* (1.6), molecular weight (425.9 Daltons) and dose (10 mg per day) [1]. Its physicochemical properties suggest that transdermal drug delivery would be beneficial, but the challenging aspect in transdermal delivery route is permeation through stratum corneum [2]. 

Several technological approaches have been attempted to overcome this challenge. They are physical approaches, chemical approaches, formulation approaches, and so forth [3]. 

Chemical approach is the most widely implemented. Chemical enhancers based on their physico-chemical properties enhance permeation through the skin by different mechanisms such as creating diffusion pathways for drug by extracting lipids from the skin, disrupting highly ordered lipid lamellae by partitioning into lipid bilayers, fluidization of lipids, and enhancing the thermodynamic activity of drugs in the formulation. Based on chemical structures of the enhancers they have been classified into water, hydrocarbons, alcohols, acids, amines, amides, esters, surfactants, sulfoxides, terpenes, lipids, and so forth [4–7].

In the present study, enhancers from different classes have been selected based on the literature reported. The enhancers selected are citric acid (organic acid), oleic acid (fatty acid), isopropyl myristate (ester), transcutol (glycol), *n*-methyl pyrrolidone (pyrrolidones), di-methyl sulfoxide (sulfoxides), tween-20 (non-Ionic surfactant), and *n*-lauroyl sarcosine (anionic surfactant). Each enhancer effect at two different concentrations (with respect to the enhancer reported literature) was evaluated by permeation studies in Carbopol gel formulation using Keshary-Chien diffusion cell. Chemical enhancers have been reported to cause skin irritation, so primary skin irritation studies have been performed and histopathological studies for optimized enhancer.

The aim of the present study is to optimize the best chemical enhancer for transdermal permeation of AH.

## 2. Experimental Details

### 2.1. Materials

Alfuzosin hydrochloride (AH) was obtained as a gift sample from Dr. Reddy's Laboratories Ltd (Hyderabad, India). Acrypol-980 was purchased from Corel Pharma Ltd (Ahmedabad, India). Citric acid, oleic acid, isopropyl myristate, dimethyl sulfoxide, *n*-methyl pyrrolidone, *n*-lauroyl sarcosine, transcutol, tween-20, propanol, glycerin and Triethanolamine were purchased from SD Fine-Chem. Ltd., India.

### 2.2. Preparation of Gels

Appropriate quantities of propanol, glycerin, and water given in Table 1 were mixed together, and the mixture was divided into two equal parts. Acrypol 980 (2%) was added to one part and soaked for 1 hour. Drug AH (1%) was added to the other part, and this solution was added to acrypol solution. Appropriate amounts of Triethanolamine were added to the solution and mixed until the gel was formed. This was considered as a base gel and taken as control. Chemical enhancer in appropriate concentration according to Table 2 was added to the acrypol solution, to which drug solution was added and allowed to gel by adding Triethanolamine.

### 2.3. Solubility Studies

Saturated solubility of AH was evaluated by adding an excess of drug to 10 mL of propanol, glycerin, and water mixture (5 : 5 : 90) including appropriate quantity of chemical enhancer. The suspension was shaken using a rotary shaker for 24 hr at room temperature; later it was centrifuged for 15 min at 3000 rpm, filtered, and diluted with the vehicle. AH concentration was analyzed by UV-VIS double-beam spectrophotometer (Chemito Spectrascan UV2600, India) at 245 nm. The effect of chemical enhancer was determined by enhancement ratio which was calculated by dividing the solubility of AH in chemical enhancer to the solubility in control (no enhancer).

### 2.4. In Vitro Diffusion Studies

Diffusion studies of the formulations were performed using locally fabricated Keshary-Chien diffusion cell of receptor volume 20 mL. The dialysis membrane was mounted between the donor and receptor compartments. 500 mg of gel formulation was applied uniformly to the dialysis membrane and the compartment clamped together. The receptor compartment was filled with phosphate buffer saline pH 7.4, and the hydrodynamics in the receptor compartment were maintained by stirring with a magnetic bead. At predetermined time intervals 1 mL of samples was withdrawn, and an equal volume of buffer was replaced. The samples were analysed after appropriate dilution for drug content spectrophotometrically at 245 nm.

AH release rate, *k*, was determined from the slope of the amount of drug released per unit area versus the square root of time [8].

### 2.5. Ex Vivo Permeation Studies

The experimental protocol was approved by the institutional animal ethics committee (IAEC).

Male Wistar rats (150–180 g) were sacrificed by excessive ether anesthesia, and abdominal hair was removed using an animal hair clipper (Aesculap, Germany). Skin was excised and observed for any cuts/wounds. The fat adhering dermis was removed using a scalpel, and it was washed under tap water. The skin was stored at −20°C and used within a week.

Locally fabricated Keshary-Chien diffusion cells with area 4.9 cm^2^ and 20 mL receptor volume were used for permeation studies. The thawed rat skin was mounted onto diffusion cell such that stratum corneum was facing donor compartment and dermis was in constant contact with receptor solution. 500 mg of gel was applied to the stratum corneum, and the hydrodynamics in the receptor compartment were maintained by stirring on magnetic stirrer at 600 rpm (Remi Equipments Ltd). 1 mL of sample was withdrawn at predetermined time intervals for 24 hrs, and drug content was analyzed by UV-VIS double-beam spectrophotometer (Chemito spectrascan UV2600, India) at 245 nm. 

After 24 hr study drug retained in the skin was determined. For skin content studies, after study the skin was removed, washed with methanol, and homogenized. The mixture was centrifuged at 7000 rpm for 30 min, filtered, and analysed for drug content spectrophotometrically at 245 nm.

### 2.6. Skin Irritation Studies

The institutional animal ethical committee approved the experimental protocol. A primary skin irritation test was performed since skin is the vital organ through which the drug is transported. The test was carried out on three healthy rabbits weighing between 1.5 and 2 kg. The test was conducted on an unbraided skin of rabbits. Before placing the formulations, the unbraided skin was cleaned with rectified spirit. The control formulation was placed on the left dorsal surface of each rabbit, whereas the test formulation (with drug and chemical enhancer) was placed on the right dorsal surface of the same rabbits, and the other rabbit was kept as control. The formulations were removed after 24 h, and the skin was examined for erythema/edema. 

### 2.7. Histopathological Studies

Histopathological studies were conducted according to the protocol approved by the institutional animal ethical committee (IAEC). The control gel formulations (placebo) and optimized gel formulations (with chemical enhancer) were applied to Wistar rats (with hair shaven at application sites) for 6 hrs. Then the animal was sacrificed, and skin was excised and stored at 50% neutral formalin solution. It was further subjected to histological processing such as dehydration and rehydration with alcohols, staining with haematoxylin-eosin dye, paraffin blocks, and slide preparation. H & E slides were evaluated using dark-light microscope by a blinded assessor.

### 2.8. Data Analysis

The cumulative amount permeated in 24 hrs (*Q*_24_) was calculated from permeation studies. Flux (*J*_ss_) was calculated from the slope of the curve on plotting *Q*_24_ versus time, and X-intercept of a straight-line portion of the curve is lag time. Flux divided by the donor concentration resulted in an apparent permeability coefficient (*Kp*). Means and standard deviation were calculated using Microsoft Excel 2003. The experiments were performed in triplicate (*n* = 3), and data were subjected to one-way ANOVA at a significance level of *P* ≤ 0.05 using MINITAB 16 software (Minitab Inc., PA, USA).

## 3. Results and Discussions

The present investigation was carried out to optimize the chemical penetration enhancers for delivering effective therapeutic amounts of AH through the skin. AH base gel (control) was prepared according to the composition in Table 1. Chemical penetration enhancers were incorporated in the gel according to the concentration of the enhancers given in Table 2.

Solubility of AH in a solvent mixture (propanol : glycerine : water (5 : 5 : 90)) of control gel was evaluated and represented as unity. Enhancement of solubility of AH by different enhancers is listed in Table 3. Maximum enhancement of solubility was shown by oleic acid 2.5% (42.92 ± 0.65 mg/mL) by 1.83-fold, followed by transcutol 20% (42.60 ± 1.15 mg/mL) by 1.82-fold, *n*-methyl pyrrolidone 10% (40.61 ± 1.32 mg/mL) by 1.73-fold, transcutol 10% (38.27 ± 1.03 mg/mL) by 1.63-fold, dimethyl sulfoxide 5% (35.88 ± 0.87 mg/mL) by 1.53-fold, *n*-lauroyl sarcosine 2% (35.32 ± 1.02 mg/mL) by 1.51-fold, isopropyl myristate 10% (33.53 ± 1.10 mg/mL) by 1.43-fold, and *n*-methyl pyrrolidone 5% (32.74 ± 1.15 mg/mL) by 1.40-fold. Solubility was decreased by tween-20 (1%) when compared with control, but showed a maximum release rate (396.36 ± 0.53 *μ*g/cm^2^/hr^1/2^) through dialysis membrane. Oleic acid 2.5% which showed maximum enhancement of solubility decreased the release rate (58.95 ± 0.39 *μ*g/cm^2^/hr^1/2^) when compared with control.

**Figure 1:**
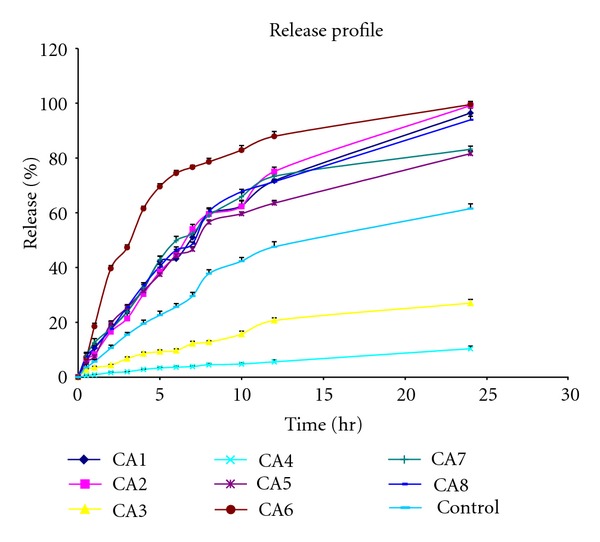
Release profile of formulations CA1 to CA8 in comparison to control through dialysis membrane.

**Figure 2:**
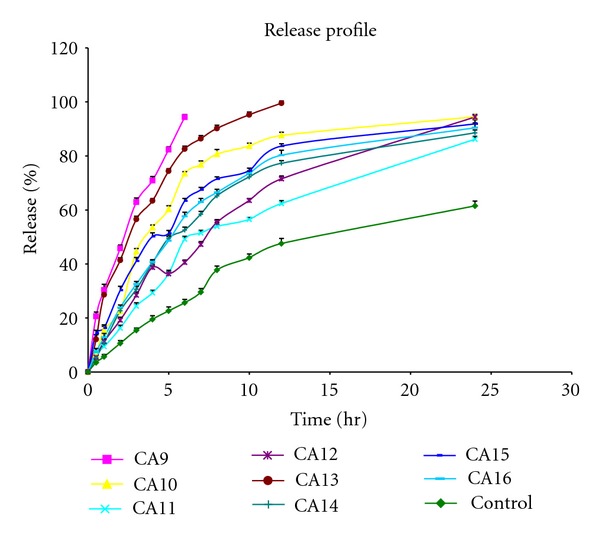
Release profile of formulations CA9 to CA16 in comparison to control through dialysis membrane.

The diffusion studies were performed on locally fabricated keshary-chein diffusion cell through dialysis membrane. The percentage release in 24 hrs by different enhancers when compared with control is shown in Figures 1 and 2. The release rate of these formulations is given in Table 3. Formulation CA9 containing tween-20 (1%) showed the maximum release (396.36 ± 0.53 *μ*g/cm^2^/hr^1/2^) in 6 hrs only, and CA13 formulation containing dimethyl sulfoxide 5% showed the maximum release (324.07 ± 0.52 *μ*g/cm^2^/hr^1/2^) in 12 hrs only. Formulations CA3 and CA4 containing oleic acid 2.5% and 5%, respectively, decreased release (58.95 ± 0.39 *μ*g/cm^2^/hr^1/2^, 20.81 ± 0.28 *μ*g/cm^2^/hr^1/2^) of drug when compared to control. Each enhancer was formulated in two different concentrations, and with an increase in concentration, increase in release rate was observed except with oleic acid, tween-20, and dimethyl sulfoxide where release rate was decreased with increase in concentration.

**Figure 3:**
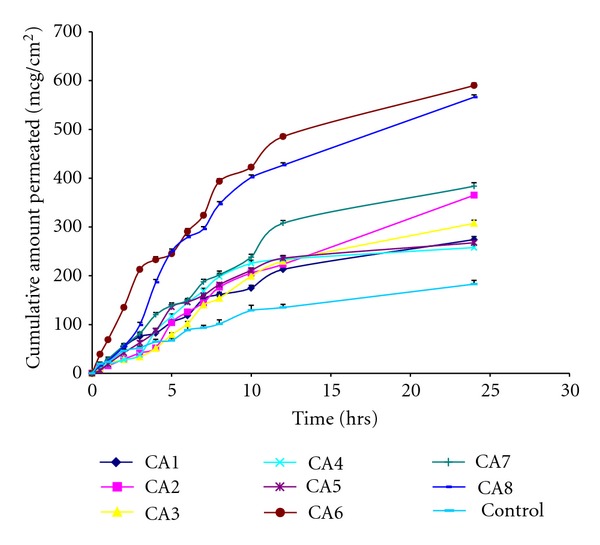
Permeation profile of formulations CA1 to CA8 in comparison with control through the rat abdominal skin.

**Figure 4:**
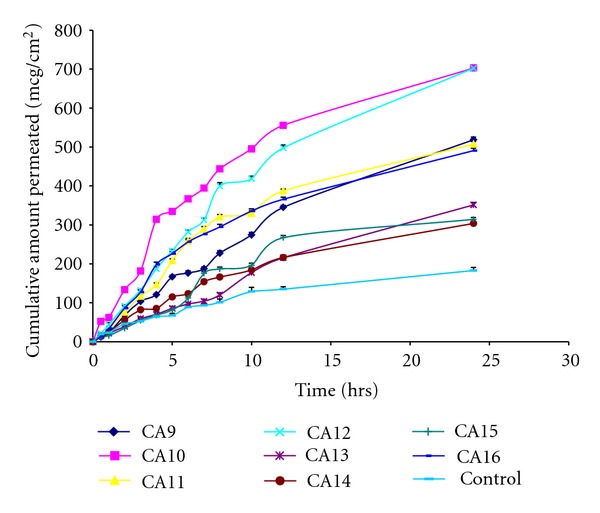
Permeation profile of formulations CA9 to CA16 in comparison with control through the rat abdominal skin.

Permeation studies using rat abdominal skin were performed and the parameters calculated (Table 4). Permeation profile is shown in Figures 3 and 4 in comparison with control. Maximum cumulative amount permeated was seen with formulations CA12 (702.28 ± 6.97 *μ*g/cm^2^), and CA10 (702.74 ± 7.49 *μ*g/cm^2^) containing transcutol 20% and tween-20 (2%), respectively, followed by isopropyl myristate 10% (589.89 ± 5.05 *μ*g/cm^2^), *n*-lauroyl sarcosine 2% (566.02 ± 4.71 *μ*g/cm^2^), tween-20 (1%) (518.65 ± 6.69 *μ*g/cm^2^), and transcutol 10% (507.26 ± 6.73 *μ*g/cm^2^). 

Transcutol 20% showed, the lowest lag time of 0.13 ± 0.05 hr followed by tween-20 (2%) (0.30 ± 0.20 hr), *n*-methyl pyrrolidone 10% (0.43 ± 0.15 hr), transcutol 10% (0.46 ± 0.15 hr), and dimethyl sulfoxide 5% (0.50 ± 0.10 hr) when compared with control (2.96 ± 0.35 hr). After 24 hrs of study, drug retained in skin was determined, and the lowest skin content was obtained with oleic acid 2.5% (342.33 ± 05.30 *μ*g/gm), transcutol 20% (355.93 ± 8.60 *μ*g/gm), dimethyl sulfoxide 10% (561.93 ± 9.26 *μ*g/gm), and *n*-lauroyl sarcosine 2% (567.82 ± 11.96 *μ*g/gm) when compared with control (1246.79 ± 10.63 *μ*g/gm). The flux was enhanced by enhancers when compared with control (7.59 ± 0.27 *μ*g/cm^2^/hr) by 3.94-fold by transcutol 20% (31.08 ± 0.21 *μ*g/cm^2^/hr) followed by 3.85-fold by tween-20 (2%). All the enhancers enhanced the permeation of AH when compared to control. Even oleic acid which decreased the release rate enhanced permeation of AH through rat skin.

Skin-irritation studies were performed on rabbits with higher concentration of each enhancer used, according to the protocol approved by institutional animal ethical committee. The effect has been graded based on the extent of erythema caused as 0—no erythema, 1—very slight erythema (barely perceptible), 2—well-defined erythema, and 3—moderate-to-severe erythema [9], and the results are given in Table 5. 

### 3.1. Effect of Chemical Enhancers

#### 3.1.1. Effect of Organic Acid

Citric acid 1% and 2.5% increased the solubility of AH by 1.13- and 1.25-fold, respectively. Increase in concentration increased release rate through dialysis membrane. Permeation also increased linearly with concentration (*P* < 0.000). No signs of skin irritation were observed. AH permeability through rat skin was enhanced by 2.05- and 1.51-fold by citric acid 2.5%, and 1%, respectively. Citric acid 1% enhanced permeation of indapamide by 3.47, fold across the rat abdominal skin by formation of an ion pair [10].

#### 3.1.2. Effect of Fatty Acids and Esters

Fatty acid and oleic acid at concentrations 2.5% and 5% were used. Maximum solubility of AH was obtained with 2.5% but the release rate was decreased. Permeability was enhanced, but with an increase in concentration, enhancement decreased (*P* < 0.000). Maximum lag time (2.70 ± 0.20 hr with 2.5% and 2.33 ± 0.15 hr with 5%) was seen with oleic acid. The decrease in release rate when compared to control can be due to the mechanism of fatty acids, such as partitioning into lipid bilayers, that is, stratum corneum and forming lipophilic complexes with drugs [3]. The release rate was measured through the dialysis membrane where it is not impregnated with lipids, and hence even though the solubility is increased, there is a decrease in release rate. The inverse relationship of concentration and enhancement was observed which has also been reported with meloxicam gel [11]. Pretreatment of tissue with fatty acids has been reported to decrease the lag time and enhance drug retainment in skin [12]. In the experimental condition pretreatment was not done, and hence increase in lag time might have resulted. 

Incorporation of the most widely studied ester isopropyl myristate at 5% and 10% showed enhanced solubility, release rate through a dialysis membrane, and permeation across the rat abdominal skin. The enhancement was linear with concentration (*P* < 0.000). They are known to enhance permeation by partitioning themselves in the ordered lipid domains of the stratum corneum [7, 13]. A similar effect was also observed with nicorandil [14] and diclofenac sodium [8] wherein shortening of lag time was also reported. In the present study also similar effect of decreased lag time (1.76 ± 0.15 hr with 5% and 0.76 ± 0.15 hr with 10%) with an increase in concentration was seen. No sign of skin irritation was observed with oleic acid and isopropyl myristate.

#### 3.1.3. Effect of Surfactants

Anionic and nonionic surfactants are more widely studied [3] in evaluating penetration enhancement abilities. In the present study *n*-lauroyl sarcosine, an anionic surfactant, and tween-20, a nonionic surfactant have been studied at two concentrations 1% and 2%. With anionic surfactant a linear relation was observed between concentration and solubility, release rate and permeation through the rat abdominal skin (*P* < 0.000). Well-defined erythema was observed when tested on rabbit skin. 

With nonionic surfactant tween-20, with 1%, the solubility decreased when compared with control, but with 2% solubility was enhanced by 1.22 fold. The release rate was increased by 1% such that the maximum amount of AH diffused within 6 hrs but as the concentration increased the release rate decreased. A linear effect was observed with concentration and permeation through the rat abdominal skin (*P* < 0.000). The results were in accordance with studies reported with meloxicam gel where permeability through IPM-saturated cellulose membrane decreased as a tween-20 increased, and no changes in overall permeability effect through human cadaver skin were observed [11]. Tween-20 caused very slight erythema when tested on rabbit skin. In general surfactants act by swelling of keratinocytes, disruption of lamellar structure of lipids, denaturation of keratin, dissolution of skin lipids, and fluidization of lipid bilayers [15].

#### 3.1.4. Effect of Glycols

The effect of diethyleneglycol monoethylether (transcutol) on the permeation of AH was investigated. Transcutol is known as a powerful solubilising agent and is an attractive penetration enhancer due to its nontoxicity, miscibility with polar and nonpolar solvents and biocompatibility with the skin [16]. In the present study transcutol enhanced solubility by 1.82 fold and release rate was also enhanced as the percentage increased. Permeation was enhanced by 3.94 fold, and it was significant with increase in concentration (*P* < 0.000). When compared with other enhancers, maximum solubility and maximum permeation through rat abdominal skin were observed. Very slight erythema of rabbit's skin was observed from skin irritation studies. The linear relation of transcutol percentage and permeation was also observed in clonazepam transdermal permeation where the enhancement is reported due to solubilising properties of transcutol and its ability to increase drug cutaneous retention [16].

#### 3.1.5. Effect of Sulphoxides

Incorporation of dimethyl sulfoxide 5% enhanced the solubility, release rate, and permeability coefficient whereas with increasing the concentration to 10%, the effect decreased (*P* < 0.001). The similar effect of decreased permeation with 10% dimethyl sulfoxide was observed with transdermal delivery of aspirin [17]. Dimethyl sulfoxide is known to enhance permeation at concentrations exceeding 60%, and it is also known to cause erythema at higher concentrations [18]. So, lesser concentrations were selected for investigating its effect. 5% were found to enhance permeation better than 10%, and it caused well-defined erythema. Sulfoxides enhance permeation by different mechanisms such as extraction of skin lipids and denaturation of stratum corneum proteins [19].

#### 3.1.6. Effect of Pyrrolidones

*N*-methyl pyrrolidone, the most extensively investigated compound of this group, was studied in two concentrations 5% and 10%. Solubility, release rate, and permeability coefficient were enhanced as the percentage increased (*P* < 0.000). Moderate-to-severe erythema was observed even with lower concentration. Pyrrolidones are known to enhance permeation by partitioning into the stratum corneum and altering the solvent nature of the membrane [20].

Among the different chemical enhancers used formulation CA12 containing transcutol 20% was optimized as maximum enhancement of permeation by 3.94 fold was obtained.

**Figure 5:**
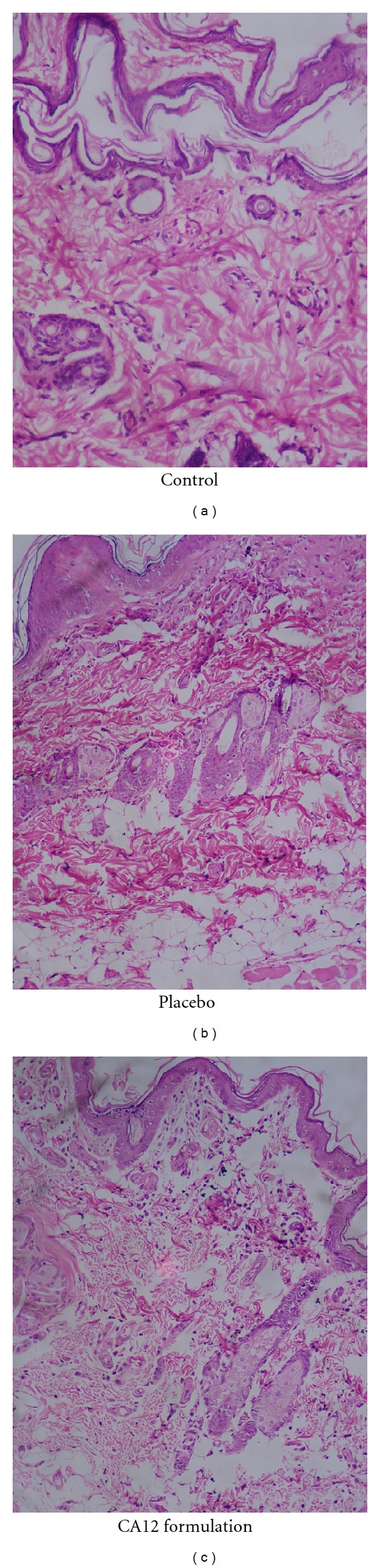
Histological slides of control (without any formulation), placebo (control gel), and CA12 (optimised chemical enhancer formulation).

Histopathology studies of the optimized enhanced formulation (CA12) containing transcutol 20% and placebo gel (control without enhancer) were performed and compared with control (Figure 5) according to the protocol approved by institutional animal ethical committee. When compared with control H & E slides of placebo gel showed severe congestion, hemorrhage, and degeneration in dermis with moderate-to-severe inflammatory changes and edema in the epidermis. Slides of optimized formulation showed congestion, degeneration, and inflammatory cells predominantly mononuclear cell infiltration in the dermis.

Changes in the skin treated with placebo gel when compared with control showed that carbopol permeates or enhances permeation. With CA12 formulation major changes in the dermis were observed which can be attributed to the action of transcutol.

## 4. Conclusion

In transdermal delivery, due to excellent barrier function of skin, the choice of an efficient chemical penetration enhancer is important. In the present study among the various enhancers evaluated transcutol 20% showed maximum permeation and the enhancement appears to be related to solubilising properties of transcutol which is shown by enhancement of AH solubility by 1.82 fold. Histopathological studies further conformed its enhancement by degenerative changes in the dermis.

## Figures and Tables

**Table 1:** Composition of base gel (control) formulation.

Ingredient	%w/w
Drug (Alfuzosin hydrochloride)	1%
Carbopol-980	2%
Propanol	5%
Glycerin	5%
Triethanolamine	q.s.
Methylparaben and propyl paraben	q.s.
Distilled water upto	10 g

**Table 2:** Formulations with different chemical enhancers and respective concentrations used.

Formulation Code	Enhancer	Concentration
CA1	Citric acid	1%
CA2	Citric acid	2.5%
CA3	Oleic acid	2.5%
CA4	Oleic acid	5%
CA5	Isopropylmyristate	5%
CA6	Isopropylmyristate	10%
CA7	*N*-lauroyl sarcosine	1%
CA8	*N*-lauroyl sarcosine	2%
CA9	Tween-20	1%
CA10	Tween-20	2%
CA11	Transcutol	10%
CA12	Transcutol	20%
CA13	Dimethyl sulfoxide	5%
CA14	Dimethyl sulfoxide	10%
CA15	*N*-methylpyrrolidone	5%
CA16	*N*-methylpyrrolidone	10%

**Table 3:** Solubility of AH in different chemical enhancers and release rate of formulations through dialysis membrane.

Formulation code	Solubility^a^ (mg/mL)	ER_sol_^b^	Release rate (*μ*g/cm^2^/hr^1/2^)
Control	23.34 ± 1.07	1	143.41 ± 0.10
CA1	26.42 ± 1.05	1.13	223.37 ± 0.21
CA2	29.34 ± 1.12	1.25	230.99 ± 0.05
CA3	42.92 ± 0.65	1.83	58.95 ± 0.39
CA4	27.65 ± 1.02	1.18	20.81 ± 0.28
CA5	25.44 ± 1.08	1.08	194.75 ± 0.21
CA6	33.53 ± 1.10	1.43	241.47 ± 0.08
CA7	30.86 ± 1.43	1.32	205.35 ± 0.43
CA8	35.32 ± 1.02	1.51	219.79 ± 0.25
CA9	20.92 ± 1.12	0.89	396.36 ± 0.53
CA10	28.67 ± 1.05	1.22	254.44 ± 0.18
CA11	38.27 ± 1.03	1.63	197.61 ± 0.37
CA12	42.60 ± 1.15	1.82	214.94 ± 0.25
CA13	35.88 ± 0.87	1.53	324.07 ± 0.52
CA14	26.90 ± 0.93	1.15	221.29 ± 0.32
CA15	32.74 ± 1.15	1.40	219.26 ± 0.24
CA16	40.61 ± 1.32	1.73	224.50 ± 0.29

^a^Solubility is the solubility of AH in the hydrogel solvent mixture at 25°C.  ^b^ER_sol_ is enhancement ratio of AH solubility over control solubility. Values represent mean ± S.D (*n* = 3).

**Table 4:** Permeation parameters of AH formulations.

Formulation code	*Q*_24_ (*μ*g/cm^2^)	Flux (*μ*g/cm^2^/hr)	Permeability coefficient (×10^−03^) (cm/hr)	Lag time (hr)	Skin content (*μ*g/gm)	ER
Control	182.84 ± 7.81	7.59 ± 0.27	1.51 ± 0.05	2.96 ± 0.35	1246.79 ± 10.63	1
CA1	274.17 ± 6.17	11.91 ± 0.16	2.38 ± 0.03	1.06 ± 0.15	981.20 ± 05.65	1.51
CA2	365.05 ± 4.78	16.16 ± 0.22	3.23 ± 0.04	2.26 ± 0.15	754.61 ± 16.47	2.05
CA3	307.43 ± 6.40	14.24 ± 0.19	2.84 ± 0.03	2.70 ± 0.20	342.33 ± 05.30	1.80
CA4	257.73 ± 6.02	12.56 ± 0.19	2.51 ± 0.03	2.33 ± 0.15	1032.36 ± 07.93	1.59
CA5	267.59 ± 6.66	12.51 ± 0.19	2.50 ± 0.03	1.76 ± 0.15	1133.28 ± 11.33	1.58
CA6	589.89 ± 5.05	25.34 ± 0.09	5.06 ± 0.01	0.76 ± 0.15	955.63 ± 09.41	3.21
CA7	383.55 ± 7.03	17.08 ± 0.21	3.41 ± 0.04	0.80 ± 0.10	676.34 ± 09.68	2.16
CA8	566.02 ± 4.71	25.88 ± 0.08	5.17 ± 0.01	1.10 ± 0.20	567.82 ± 11.96	3.28
CA9	518.65 ± 6.69	22.39 ± 0.20	4.47 ± 0.04	1.23 ± 0.15	713.82 ± 12.33	2.84
CA10	702.74 ± 7.49	30.38 ± 0.18	6.07 ± 0.03	0.30 ± 0.20	592.20 ± 08.54	3.85
CA11	507.26 ± 6.73	22.67 ± 0.18	4.53 ± 0.03	0.46 ± 0.15	651.33 ± 09.00	2.87
CA12	702.28 ± 6.97	31.08 ± 0.21	6.21 ± 0.04	0.13 ± 0.05	355.93 ± 08.60	3.94
CA13	351.39 ± 6.95	14.87 ± 0.14	2.97 ± 0.02	0.50 ± 0.10	877.00 ± 08.28	1.88
CA14	303.85 ± 6.08	12.99 ± 0.15	2.59 ± 0.03	0.83 ± 0.15	561.93 ± 09.26	1.64
CA15	314.25 ± 4.80	14.79 ± 0.09	2.95 ± 0.01	1.33 ± 0.15	875.97 ± 08.61	1.87
CA16	490.75 ± 6.04	21.38 ± 0.08	4.27 ± 0.01	0.43 ± 0.15	687.58 ± 08.30	2.71

*Q*_24_ is the cumulative amount permeated in 24 hrs; ER is an enhancement ratio of the flux of chemical enhancers over control. Values represent mean ± S.D (*n* = 3).

**Table 5:** Skin irritation effect of different chemical enhancers.

Chemical enhancer with concentration	Skin irritation
Citric acid (2.5%)	0
Oleic acid (5%)	0
Isopropyl myristate (10%)	0
*N*-lauroyl sarcosine (2%)	2
Tween-20 (2%)	1
Transcutol (20%)	1
Dimethyl sulfoxide (10%)	2
*N*-methyl pyrrolidone (10%)	3
